# Innovation to Impact: Introduction to the Special Issue on Evidence from the Teen Pregnancy Prevention Program Experiment with Innovation

**DOI:** 10.1007/s11121-023-01620-3

**Published:** 2023-12-04

**Authors:** Elizabeth Laferriere, Nicole Bennett, Emily Forrester, Tara Rice, Jaclyn Ruiz

**Affiliations:** 1 Office of Population Affairs, Washington, DC USA; 2 Laerdal Labs, Washington, DC USA; 3 Mathematica, Princeton, NJ USA

**Keywords:** Innovation, Adolescent sexual health, Teen Pregnancy Prevention, Grants

## Abstract

Congress has provided funding for the federal Teen Pregnancy Prevention (TPP) Program since 2009 for spending beginning in Fiscal Year 2010. Designated TPP programs develop, test, and evaluate innovations for reducing teen pregnancy, teen pregnancy disparities, and associated risk factors and for promoting positive youth development. Since its inception, the TPP Program has experimented with multiple uniquely structured cohorts of innovation and demonstration projects, producing critical insights into equitable and effective public health innovation while also serving as a highly productive contributor of evidence-based, TPP innovations for scale. This article briefly documents the innovation history of the TPP Program and its iterations in response to the shifting needs of the field. We then synthesize findings from the fifteen TPP innovators published in this special edition. We highlight emergent priorities of the TPP Program informed by this federal experiment in rigorous adolescent sexual health innovation development, testing, evaluation, and scaling and conclude by discussing how the TPP program adapted and refined its approach for fostering dynamic innovation-to-scale projects over time.

Innovation serves a critical role in the federal government’s quest to solve the nation’s most complex and dynamic challenges, as well as meet its commitment to provide high quality, effective, and responsible programs for its citizens (Partnership for Public Service, 2019). The United States federal government has consistently catalyzed innovation throughout its 250-year history. From laying the foundation for Electronic Health Records (EHRs) to the Global Positioning System to entrepreneurial early education technology, government-backed innovation comes in many facets, and has shaped the trajectory of critical industries and social programs (Mazzucato, 2013; Metz, 2023; OECD, Facets of Innovation). One prominent example of government-backed innovation includes *Tiered Grant Designs*, in which government offices release funding for both innovation—or the development of novel, untested practices and their rigorous evaluation—as well as the replication of those approaches proven effective (CRS, 2022; Executive Office of the President, 2015; Government Accountability Office, 2016; Haskins & Margolis, 2014). The Teen Pregnancy Prevention Program (TPP Program), administered by the Office of Population Affairs (OPA), which includes the Office of Adolescent Health (OAH), in the US Department of Health and Human Services (HHS), is one such tiered program and a promising example of innovation within the federal framework.

This supplemental issue of *Prevention Science* showcases 15 original articles from four “innovation and demonstration” TPP cohorts that developed, tested, and evaluated public health innovations between 2015 and 2023. Together, these articles expand the field’s understanding of youth-centered approaches to advance adolescent sexual health; disseminate original empirical research and impact findings; share promising strategies regarding innovation design, development, and adaptation; and discuss evaluation readiness across innovation stages.

This introductory paper serves as the contextual framing for this special issue. We outline the short history but rapidly evolving structure of TPP Program innovation. We illuminate emergent themes from this diverse collection of articles written by a subsection of our growing innovation community. We conclude by reflecting on the broader contributions of the program to the greater field of adolescent sexual health and positive youth development, as well as to our own federal model for fostering dynamic innovation-to-scale projects.

## Innovation as an Essential Driver of Health Equity

While significant progress has been made to reduce teen pregnancy rates, disparities persist in terms of geography, urbanicity, race and ethnicity, and among historically “underserved” populations (Exec. Order No. 13985, 2021), such as those youth impacted by the juvenile justice and/or child welfare systems, LGBTQ + youth, or youth experiencing homelessness (Brindis et al., 2020; CDC, 2019; CDC, 2020; CDC, 2021; Finigan-Carr et al., 2021; Hamilton et al., 2016; James, 2009; Martin et al., 2022; Sedlak & Bruce, 2010). Embracing innovation presents an opportunity for the federal government and our community partners to tackle the complex interplay of social determinants of health (SDOH), including structural racism, and promote positive adolescent sexual and reproductive health outcomes, ultimately driving toward health equity. OPA considers innovation to be about both outcomes and processes. One may refer to the global design company IDEO's (undated, para. 6–7) definition of innovation as: “the ability to generate and execute new ideas—incremental, evolutionary, or revolutionary—and it starts with creativity. Creativity is the ability to look past the obvious—to transcend traditional ways of seeing the world to create something new.”

## Innovation in the Federal Teen Pregnancy Prevention (TPP) Program

The TPP Program’s congressional authorization language has been in the HHS appropriations act since Fiscal Year 2010 (*see, e.g.*, Division H, Title II of the Consolidated Appropriations Act, 2023 (Public Law No. 117–328)) and mandates the following:

“$101,000,000 shall be for making competitive contracts and grants to public and private entities to fund medically accurate and age appropriate programs that reduce teen pregnancy and for the Federal costs associated with administering and evaluating such contracts and grants, of which not more than 10 percent of the available funds shall be for training and technical assistance, evaluation, outreach, and additional program support activities, and of the remaining amount 75 percent shall be for replicating programs that have been proven effective through rigorous evaluation to reduce teenage pregnancy, behavioral risk factors underlying teenage pregnancy, or other associated risk factors [i.e., Tier 1 Replication], and *25 percent shall be available for research and demonstration grants to develop, replicate, refine, and test additional models and innovative strategies for preventing teenage pregnancy [Tier 2 Innovation and Evaluation*].” (Emphasis added)

The TPP Program is a national, tiered, and evidence-based program that funds diverse organizations working to reach adolescents to improve sexual and reproductive health outcomes and promote positive youth development. It strives to provide adolescents with “the support, confidence and resources to thrive, be healthy, and realize their full potential” (OPA, 2023a Notices of Funding Opportunity (NOFOs); Ross et al., 2020). The program is administered by the Office of Population Affairs (OPA), which includes the Office of Adolescent Health (OAH), within the U.S. Department of Health and Human Services (HHS) Office of the Assistant Secretary for Health (OASH). OPA provides grant funding through a competitive process to organizations in communities across the country.

As a two-tiered initiative, the TPP Program represented a major innovative development in grants structure. It invests in both the scaled implementation of effective programs (i.e., Tier 1) and provides funding to develop, refine, and test additional models and innovative strategies to prevent unintended teen pregnancy, sexually transmitted infections (STIs), behavioral risk factors underlying teen pregnancy, or other associated risk factors (i.e., Tier 2) (OPA, 2023a, b, c). Under TPP, innovation reflects a broad spectrum of new or adapted products, programming, strategies, approaches, interventions, policies, and practices. OPA further differentiates innovations by their pathway to future scaled delivery. Some eventually become evidence-based TPP approaches or programs (i.e., EBPs), which OPA describes as “those programs that have been proven effective through rigorous evaluation to reduce teenage pregnancy, behavioral risk factors underlying teenage pregnancy, or other associated risk factors.” Others are designed as innovative practices that youth-serving organizations (such as Tier 1 projects) may employ as part of their holistic approach to improve sexual and reproductive health outcomes, promote positive youth development, and advance health equity for adolescents, their family, and communities. Such practices are still rigorously tested but are not yet well suited for the evidence list described below.

To align with the new federal movement focused on increasing funding for evidence-based policy (Congressional Research Service, 2022), in 2009, HHS created the HHS Teen Pregnancy Prevention Evidence Review (TPPER). TPPER publicly highlights the findings of the “systematic review of research on teen pregnancy prevention to identify programs with evidence of effectiveness in favorably impacting (1) teen pregnancy and sexually transmitted infections (STIs) and (2) sexual behaviors” (Forrester et al., 2023). This enables HHS to set a consistent standard for what is considered an effective teen pregnancy prevention program. The TPPER is led by the Office of the Assistant Secretary for Planning and Evaluation (ASPE) in partnership with OPA and the Administration for Children and Families.

The TPPER has always played a critical role in shaping the TPP Program (Feldman Farb & Margolis, 2016; *see Funding Opportunity Announcements*). Between 2010 and 2020, Tier 1 grantees were required to select an effective program identified by the TPPER for scaled implementation. While the TPPER was neither funded nor updated between 2018 and 2023, the TPP Program still relied heavily on the standards for rigorous evaluation and effectiveness set by the TPPER for determining which programs were eligible for scaling, and grantees frequently referred to it when researching appropriate programs. Beginning in 2020, the TPP Program broadened its expectations and allowed grantees to propose TPP programs, including TPP programs which promote positive youth development, that met NOFO requirements and TPPER standards for quality and rigor, regardless of whether the programs themselves were included on the TPPER evidence list. For example, in 2023, the TPP Program defined eligible evidence-based programs as those that “meet the criteria for the quality of an evaluation study per the criteria established in the [TPPER]”; further, it clarified that TPP implementation (Tier 1) recipients are expected to select at least one evidence-based program deemed effective in addressing *sexual risk behaviors* using TPPER evidence standards, although it may supplement this programming using programs with evidence of effectiveness on *behavioral risk factors underlying teen pregnancy* (OPA, 2023b). Still, most Tier 1 grantees continue to leverage the TPPER list as a reference point and convenient library of pre-vetted options.

TPPER has played a similarly central role in shaping Tier 2, both establishing standards for successful impact evaluations but also serving as a historical “goal post” for innovation development under the TPP Program, as innovators traditionally endeavored to develop and advance innovations that would generate evidence of effectiveness and afford them a place on the TPPER list. In recent years, recognizing that not all innovations fit the mold of TPPER effective *programs*, OPA has adapted and encouraged TPP innovations of all shapes and sizes, such as those focused on systems change or youth engagement strategies, and continues to support the evolution of TPPER toward a future that accounts for program *components*, as well as packaged programs.

Similarly, the design and structure of TPP innovation funding have also changed over time in response to the shifting needs of the field as well as to build upon successive cohorts’ learnings on how best to leverage innovation to reduce unintended teen pregnancy and create an inclusive evidence base. Table 1 details OPA’s major Tier 2 cohorts responsible for designing, testing, and evaluating innovative TPP approaches between 2010 and 2023. Several of these cohorts emerged through critical partnerships across the federal government. While these initiatives always focused on populations with the highest need, some cohorts more narrowly defined areas of investment based on identified gaps. The table outlines the unique approach taken by each major Tier 2 project type, as well as what those approaches contributed to the field and how they shaped the office’s own adaptive Tier 2 design.

**Table 1:** Evolution of the Teen Pregnancy Prevention (TPP) Program Tier 2, 2010–2023

Years^a^	Cohort	Approach and major contributions to the Tier 2 innovation pipeline
2010–2015	Impact evaluations	The Federal Government launched the two-tiered TPP Program as part of its strategic investment in evidence-based social policy and programs (Haskins & Margolis, 2014; Kappeler & Farb, 2014). These Tier 2 projects rigorously evaluated (1) interventions with preliminary evidence of effectiveness, (2) major adaptations of effective programs, or (3) entirely new innovations (Kappeler & Farb, 2014; OAH, 2010b). This first cohort was instrumental in generating a list of evidence-based TPP programs for implementation i.e., ensuring a pool of effective programs for implementation under Tier 1. Of 19 evaluations of new and innovative approaches, eight programs demonstrated positive outcomes (Feldman Farb & Margolis, 2016; Kappeler, 2016; Koh, 2014). Additionally, the experience underlined the importance of not just funding impact evaluations of existing approaches through Tier 2 but also the development of new innovations in order to ensure an adaptive, growing, and equitable evidence base
	Community-wide initiatives	OAH and the CDC Division of Reproductive Health (DRH) partnered to fund state and local organizations in designing and implementing a community-wide TPP approach that centered on five main elements: evidence-based programs, clinical services, stakeholder engagement, community mobilization, and working with diverse communities (Fuller et al., 2016; Kappeler & Farb, 2014; Mueller et al., 2017; OAH, 2010a). These projects demonstrated the value and potential for scaled, holistic TPP approaches (House et al., 2022), as well as the need for greater exploration of multi-level, collective, and ecological strategies
2015–2020^b^	Innovation intermediaries	Recognizing equity gaps in the existing pool of effective TPP programs, OAH expanded its Tier 2 investment in the exploration and development of new and innovative approaches, in anticipation that some emergent advancements would eventually demonstrate readiness for rigorous evaluation. This shift reflected a growing appreciation for a multi-pronged Tier 2 approach that funds innovation from development through to evaluation, in order to maintain an adaptive and equitable base of evidence-based approaches OAH funded two innovation intermediaries that provided funding and technical assistance support to multiple teams of innovators that developed and tested either technological or programmatic innovation (Antonishak, 2023; OAH 2015c; Wilson et al., 2023). These intermediaries served as a true catalyst of innovation in the TPP Program, and the force that instilled the necessity of participatory co-design, methods associated with design thinking/human-centered design and systems thinking, and the benefits of collaborating with grantees to foster their own unique innovation processes. Together, these intermediaries and their teams generated 27 programmatic and 11 technological innovations*. In this edition:* Antonishak et al. 2023; Huq et al. 2023; Kuo et al. 2023; Leos et al. 2023; Wilson et al. 2023
	Impact evaluations	OAH continued to invest in rigorous impact evaluations of innovative programs. During this cohort, the office encouraged projects focused on priority populations or strategies to address gaps in the evidence base at the time, including: 18–19-year-olds, males, rural, high risk, non-traditional modalities, and diverse socio-ecological levels (OAH, 2015b). Despite the discontinuation of funding, an impressive one-third of all evaluations identified evidence of effectiveness. *In this edition:* Jenner et al. 2023; Kesler et al. 2023; O’Connell et al. 2023; Ybarra et al. 2021; 2023.
	Impact evaluations on innovations for young males	The evidence base contains few effective TPP programs developed specifically with and for young men. To address that gap, OPA and CDC collaborated to fund impact evaluations and qualitative explorations of innovative approaches that specifically focus on young men between the ages of 15 and 24 (OAH, 2015a; OPA, 2019). These projects generated data that further underscored the need for new, innovative TPP approaches developed for and with young males
2018–2020	Formative evaluations	The Office of Population Affairs (OPA), which now included OAH, learned from its first cohort that newly developed innovations require great intentionality, time, and resources dedicated to rigorous testing and refinement before they are ready for an impact evaluation grant. This cohort funded formative evaluations of innovation to better prepare both innovators and interventions for future rigorous evaluation (OAH, 2018)
2020–2023	Innovation intermediaries	Innovation Networks fused the structure of innovation intermediaries with the dynamic connectivity and collective impact approaches, influenced by Collaborative Improvement and Innovation Networks (CoIIN) (Ghandour et al., 2017; Gloor & Grippa, 2018). The thirteen Innovation Networks built vast and interconnected partnerships to support innovation development pipelines within seven priority areas: access to health care, foster youth, youth with disabilities, expectant and parenting young people, caregivers, juvenile justice, and youth engagement (OPA, 2020a) The grantees reported significant success in relational indicators, demonstrating deep and meaningful connections with stakeholders related to their subject area. While they generated an impressive number of innovations, some of the challenges they experienced suggested that OPA’s innovation approach might benefit from bifurcating further the innovation development and the rigorous innovation testing stages in the future cohort. This would encourage more expert specialization as well as allow for greater movement and idea sharing across different grant networks throughout the project period. These networks generated and/or tested dozens of innovations and nearly half of those selected for 2023 impact evaluations came directly from these networks. *In this edition:* Ball et al. 2023; Colarossi et al. 2023; Hartzler-Weakley et al. 2023.
	Impact evaluations	OPA funded its smallest cohort of impact evaluations, reflecting the need to continue to invest in new ideas and provide sufficient time for testing and refining before OPA increased the number of impact evaluations. OPA encouraged evaluations of innovations that focused on: juvenile justice, foster youth, expectant and parenting youth, youth with disabilities, youth experiencing homelessness, caregivers, individual or systems-level approaches (OPA, 2020b). *In this edition:* O’Connell et al. (2023)^c^

This table does not include all Tier 2 cohorts funded through the TPP Program

^a^Although funding opportunities are tied to fiscal years, this chart displays information in calendar years to indicate years of implementation

^b^Funding for all 2015–2020 TPP grants was temporarily discontinued by the Federal Government after the third year of their project periods, although funding was restored for the fourth and fifth years following court intervention for most, but not all grantees. While the interruption negatively impacted the Tier 2 innovation teams’ ability to implement their innovation projects as planned, the compelling results of both intermediaries and impact evaluators in spite of this situation speaks to these innovators’ robust operations, resilience, and adaptive approaches, as well as the return on investment for OAH’s heavy reliance on evaluation technical assistance and standardization of evaluation plans for evaluators (CRS, 2022; Hofert et al., 2023)

^c^The MARSSI intervention in the article by O’Connell et al. (2023) received funding in both cohorts for different phases

**Contributions to the Field**

Thirteen years into its experiment, TPP innovation stands out as a leading force driving advancements in sexual health programming with and for young people and in collaboration with key federal partners. What follows are three contributions of the program overall as well as its community of innovators:

1. OPA’s innovation program is a prolific catalyst of evidence-based, equity-advancing TPP approaches for the field

Since its establishment, TPP early innovators have advanced well over one hundred independent innovations for diverse populations, modalities, settings, and more. As with any innovation program, TPP recognizes the importance of divergence and generating a large quantity of ideas with community input before ultimately narrowing to those that resonate and meet expectations of desirability, viability, and feasibility for the population of focus. OPA’s phased approach to TPP and its investment in early innovation, piloting, and rigorous evaluation has demonstrated significant results in moving promising ideas along and preparing them to meet key milestones.

One measure of success for an innovation program is the rate of successful progression, moving from early ideas to prototype to testing to evaluation and eventually being identified as evidence based. For TPP, the numbers speak for themselves. Over half (28) of all programs (52) on the evidence review were developed or tested through the TPP Program (Forrester et al, 2023; OPA, 2023c). In 2023, when the TPPER announced its first update in five years, seven of the nine new innovative programs meeting the quality and evidence standards under TPPER were developed with support from Tier 2 Innovation funding (Forrester et al, 2023). In the most recent announcement of the new 12 impact evaluations, awarded in September 2023, five (42%) came directly from OPA’s Innovation Networks (2020–2023)—including three entertainment education innovations built with and for youth in the juvenile justice system, one caregiver engagement approach, and a unique systems-level program for foster youth. In this supplement, Ball et al. (2023) detail the formative research and co-design findings which contributed to the development of the foster youth intervention accepted into the latest evaluation cohort.

The articles in this edition explore how over time Tier 2 continues to contribute to expanding a more representative and diverse base of TPP innovations, including those for youth with disabilities (Colarossi et al., 2023; Hartzler-Weakley et al., 2023), foster youth (Ball et al., 2023), rural youth (Leos et al., 2023), Native Hawaiian and Pacific Islander youth experiencing homelessness (Huq et al., 2023; Kuo et al., 2023), Native American and Indigenous youth (Begay et al., 2023; Chambers et al., 2023), Latino youth (Faccio et al., 2023), Black and Hispanic females (Jenner et al., 2023), and sexual minority girls (Ybarra et al., 2021; 2023). Kesler et al. (2023) also demonstrate that their evidence-based innovation, High School FLASH, while developed for general high school delivery, also reduces homophobic and transphobic beliefs among identified populations, furthering the conversation of additional outcome indicators and/or precursors of interest for the TPP Program. With greater diversity in evidence-based offerings, the TPP Program is far better situated to meaningfully tackle disparate outcomes across youth subpopulations that have long been a source of concern in the public health community.

2. OPA innovators co-designed alongside youth, caregivers, and community members, not just building more responsive innovations, but also empowering ongoing community action with innovation tools, methods, and mindsets

Although small in number, OPA’s innovation grantees have exceeded expectations in terms of community engagement and implementation and evaluation capacity-building. For example, from 2020 to 2023, the 13 Innovation Networks conducted 2,283 trainings for 13,565 individuals and in the process of designing and testing their innovations, engaged 27,466 young people, 9,066 parents and caregivers, and 316,424 community members (OPA, 2023d).

In this supplemental issue, Colarossi et al. (2023) and Hartzler-Weakley et al. (2023) critically examine their Innovation Network development experiences. The first paper details process findings related to facilitating a high-functioning collaborative network of multidisciplinary and community partners to advance more innovations for youth with disabilities. The second paper presents formative findings generated in partnership with professionals who serve youth with disabilities about their attitudes, preparedness to serve, current practice, and professional needs related to that service. Ball et al. (2023) present findings related to their multidisciplinary learning community which explored the conditions, challenges, and opportunities to serve foster youth at multiple levels of the social ecology. Leos et al. (2023) further outline the role of human-centered design in producing innovations for low-income and rural youth. These papers document formative research which has informed the development of promising, co-designed innovations for these specific youth populations that experience persistent adolescent sexual health disparities.

3. Our collective experiences and lessons learned have culminated in OPA’s reimagining of its TPP innovation-to-scale model and funding approach

Not to be underplayed is the significance of our innovators’ commitment to ongoing dialogue and partnership with the federal government in order to continually improve the overall structure of the TPP Program. Together, we work to shape an optimal grant structure that rises to meet the challenges and complexity at hand. Our community of grantees consistently participate in focus groups, design sessions, workshops, and more to that end. OPA staff have fostered a culture of trust, experimentation, openness to failure, and co-design; grantees have reciprocated by openly sharing with OPA their challenges, opportunities, and perspectives with respect to how to create the most effective and equity-focused federal innovation program to address adolescent sexual health. In 2021–2022, OPA hosted four interrelated workshops with grantees as well as outside experts to explore important questions about optimal structures and practices for facilitating innovation, challenges with generating early evidence, gaps in the evidence base and emergent needs from the field, and experiences with the most recent cohort of Innovation Networks. Data from these sessions as well as from dialogue with current grantees and analysis of their performance informed two important advancements for the TPP Program: (1) a fully reimagined innovation-to-scale continuum model and (2) our latest cohort (2023–2028) of further tiered innovation grants.

**Fig. 1:**
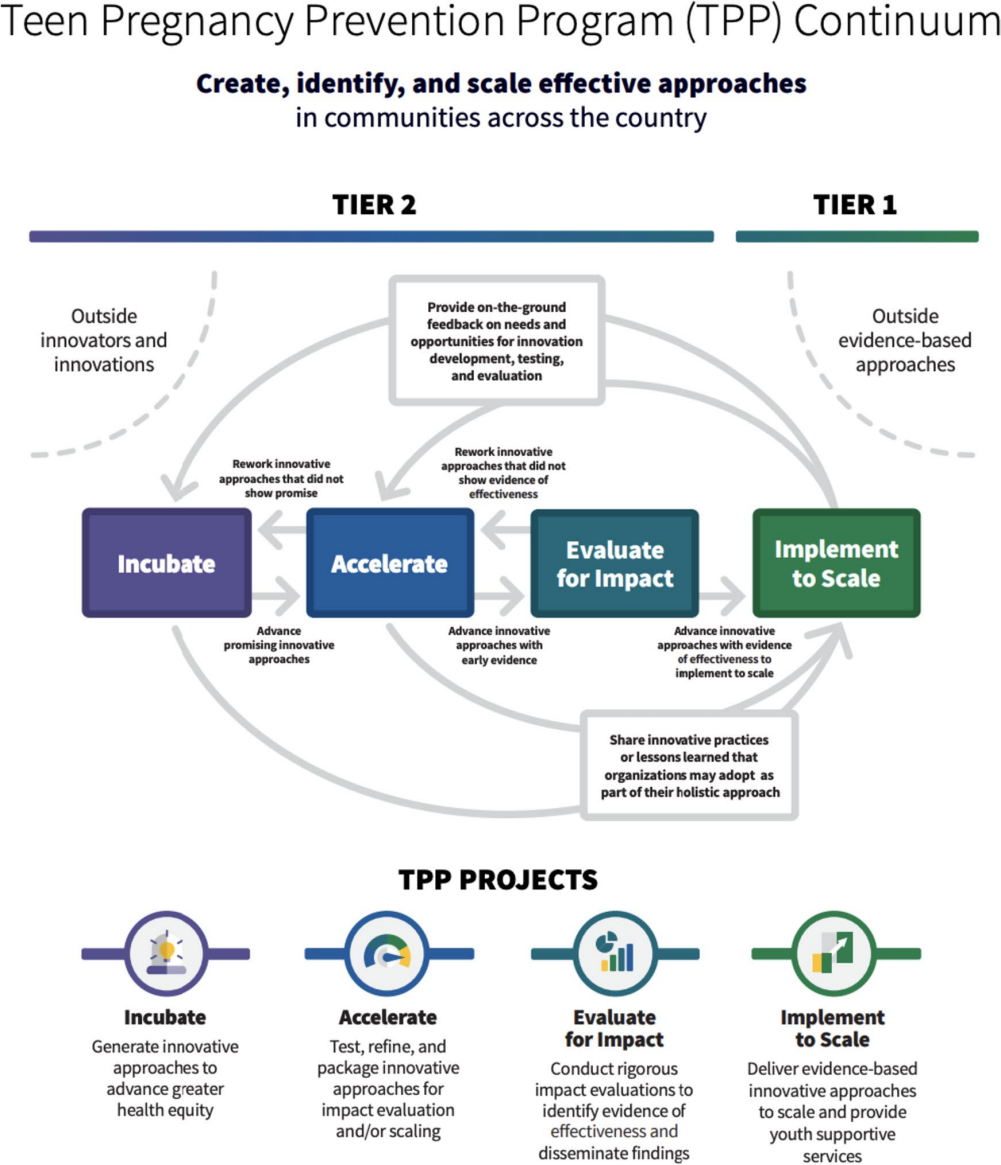
Teen Pregnancy Prevention Program continuum model (2023–2028)

In Fig. 1, our new innovation-to-scale continuum model reflects the multi-tiered nature of the program. While OPA has used visual representations of tier interactions in the past, this continuum demonstrates a new approach to thinking about the TPP Program and the processes that take place therein. First, the continuum now focuses on a dynamic representation of the multi-tiered program. Information and innovations can move in either direction, which enables the TPP Program to stay responsive, grounded, connected, and impactful. This visual was created after the series of focus groups with the community described above and underwent internal testing with OPA and key partners. The purpose of the model is to provide a visual that empowers OPA and our grantees to document and disseminate a clear and compelling story about the dynamic and universal nature of innovation, how everyone in our grant community has a role in advancing health equity by contributing to the evidence base, and that feedback loops are expected and necessary for staying relevant in a fast-changing world.

OPA’s current Tier 2 grant project funding opportunity announcement put this continuum into practice. Included in this project announcement are the Adolescent Sexual Health Innovation Hubs (“Innovation Hubs”), which are designed to be the most dynamic, phased, and equity-focused innovation grant design yet (OPA, 2023a). This new approach differentiates innovation across three, instead of two, layers: (1) incubators support teams to create innovative approaches, (2) accelerators support teams in testing, refining, and packing innovative approaches in preparation for impact evaluation, and (3) impact evaluators contribute to our growing knowledge base around what works and what does not in promoting adolescent sexual health. It also allows for greater movement of innovation teams across these approaches throughout grant periods.

Finally, the pendulum is swinging back in support of impact evaluation. Given how many innovations were developed through previous cohorts of innovation intermediaries (2015–2020) and Innovation Networks (2020–2023), OPA decided to once more increase the number of rigorous evaluation grants back from 4 in 2020 to 12 in 2023. The rigorous evaluations have evolved too, calling attention to the need for more evaluations of innovations that are non-school based such as self-administered technological interventions, short dosage interventions particularly single-touch point innovations, and community and system-level approaches.

## Future Directions

In September 2023, OPA launched its current cohort of innovators and will continue to learn and refine its innovation pipeline structure in partnership with the field. In addition, OPA must prioritize not only generating and identifying evidence-based innovations, but also increasing meaningful awareness and uptake of said innovations. We innovate not just for the sake of innovation, but to share, scale, and maintain this dynamic process, all toward improving the lives of our nation’s youth. Yet, despite having one of the most prolific innovation generators in the nation, OPA identified lower than expected awareness and uptake of innovations, through focus groups and interviews. We discovered four themes in conversation with our community: (1) grantees are interested in enhanced partnerships across Tiers 1 and 2 but do not yet feel like they currently have the capacity, tools, or the clear incentive to develop these connections; (2) many Tier 1 s believed innovation referred only to recently identified EBPs in the 2015–2020 cohort and are unaware of the diverse strategies and approaches being tested that are not yet and/or will never be EBPs under current definitions; (3) Tier 2s did not realize that Tier 1s had such low awareness of what they do, and see opportunities for investing in partnership regarding testing, implementation, and dissemination; and finally (4) to make meaningful partnership possible, OPA should leverage its position to support greater collaboration for mutual benefit. OPA will use these themes to inform a series of next steps.

First, OPA is co-designing a dissemination and awareness strategy alongside our innovator community, including those with past and current funding, but also bringing in new community voices from the TPP Tier 1 cohort as well as providers and grantees in OPA’s Title X Family Planning network. This collective project is exploring a diverse range of solutions to meet its goal, including four videos amplifying the stories of innovators with promising projects, capacity-building, guidance, and technological solutions.

Second, but no less significant, OPA recognizes the need for not just innovation grants, but also innovation community, in order to leverage our network to achieve OPA’s vision. In 2023, OPA launched its effort to increase the sense of community in sharing across grantees through two in-person convenings. The first, the Innovation Exchange, convened 50 innovator teams from TPP Tiers 1 and 2 as well as Title X for two days of peer-sharing their innovations (from mobile apps to education entertainment series to radical partnerships), soliciting feedback, building and practicing their story, and discussing the challenges of sexual and reproductive health innovation in the current environment. OPA’s second 2023 event was its Innovation Lab at the biannual Title X Conference. During this session, 14 innovators demonstrated their products, practices, and interventions for nearly 200 Title X grantees and providers as a way to more intentionally build innovation bridges across TPP and Title X.

## Conclusion

The 15 articles comprising this supplemental issue make a compelling case for the continued if not heightened national investment in strategic, intentional, and community-grounded innovation to interrupt and redress persistent adolescent sexual health disparities. The articles reflect the breadth of the TPP Program, presenting findings on system-level innovations, intervention development, adaptations, evaluation readiness, implementation science, and impacts. The concluding commentary from Hyman and Philbrick (2023) and the Partnership for Public Service reinforces this claim, arguing the necessity of innovation in federal programs for truly delivering upon the federal agency’s mission of health for all.
